# Circadian rhythm-related genes index: A predictor for HNSCC prognosis, immunotherapy efficacy, and chemosensitivity

**DOI:** 10.3389/fimmu.2023.1091218

**Published:** 2023-03-10

**Authors:** Hao Chi, Jinyan Yang, Gaoge Peng, Jinhao Zhang, Guobin Song, Xixi Xie, Zhijia Xia, Jinhui Liu, Gang Tian

**Affiliations:** ^1^ Clinical Medical College, Southwest Medical University, Luzhou, China; ^2^ School of Stomatology, Southwest Medical University, Luzhou, China; ^3^ Department of General, Visceral, and Transplant Surgery, Ludwig-Maximilians-University Munich, Munich, Germany; ^4^ Department of Gynecology, The First Affiliated Hospital of Nanjing Medical University, Nanjing, China; ^5^ Department of Laboratory Medicine, The Affiliated Hospital of Southwest Medical University, Luzhou, China

**Keywords:** HNSCC, circadian rhythm, biomarkers, tumor microenvironment, immunotherapy, prognostic signature

## Abstract

**Background:**

Head and neck squamous cell carcinoma (HNSCC) is the most common head and neck cancer and is highly aggressive and heterogeneous, leading to variable prognosis and immunotherapy outcomes. Circadian rhythm alterations in tumourigenesis are of equal importance to genetic factors and several biologic clock genes are considered to be prognostic biomarkers for various cancers. The aim of this study was to establish reliable markers based on biologic clock genes, thus providing a new perspective for assessing immunotherapy response and prognosis in patients with HNSCC.

**Methods:**

We used 502 HNSCC samples and 44 normal samples from the TCGA-HNSCC dataset as the training set. 97 samples from GSE41613 were used as an external validation set. Prognostic characteristics of circadian rhythm-related genes (CRRGs) were established by Lasso, random forest and stepwise multifactorial Cox. Multivariate analysis revealed that CRRGs characteristics were independent predictors of HNSCC, with patients in the high-risk group having a worse prognosis than those in the low-risk group. The relevance of CRRGs to the immune microenvironment and immunotherapy was assessed by an integrated algorithm.

**Results:**

6-CRRGs were considered to be strongly associated with HNSCC prognosis and a good predictor of HNSCC. The riskscore established by the 6-CRRG was found to be an independent prognostic factor for HNSCC in multifactorial analysis, with patients in the low-risk group having a higher overall survival (OS) than the high-risk group. Nomogram prediction maps constructed from clinical characteristics and riskscore had good prognostic power. Patients in the low-risk group had higher levels of immune infiltration and immune checkpoint expression and were more likely to benefit from immunotherapy.

**Conclusion:**

6-CRRGs play a key predictive role for the prognosis of HNSCC patients and can guide physicians in selecting potential responders to prioritise immunotherapy, which could facilitate further research in precision immuno-oncology.

## 1 Introduction

As the sixth most common cancer worldwide, HNSCC includes malignant lesions originating in the mouth, lips, nasopharynx, pharynx, or larynx (1). Globally, HNSCC has an incidence of more than 809,000 cases and more than 316,000 deaths, accounting for 3.6% of all cancer deaths (2). Currently, HNSCC treatment has been based on various therapeutic approaches such as surgery, chemotherapy, radiotherapy, and photodynamic therapy (3). However, the prognosis for individuals with HNSCC remains dismal, and morbidity and mortality rates are rising yearly due to the disease’s very aggressive and diverse character (4). Patients with HNSCC diagnosed at an early stage have a 60-95% chance of successful treatment by primary tumor resection combined with extensive neck debulking (5). However, most patients have advanced cancer at the time of diagnosis, with tumor metastasis and recurrence. The prognosis of the disease is strongly correlated with the TNM stage and histologic grade of HNSCC, which serve as the main foundation for several treatment choices, including prognostic classification, immunotherapy, radiation, and chemotherapy (6–10). However, the prognosis of HNSCC patients based on conventional clinicopathological staging may not be totally accurate since people with the same clinical stage may have varied clinicopathologic characteristics (9, 11). Therefore, to forecast the prognosis of HNSCC patients and direct tailored treatment, new prognostic biomarkers and molecular targets must be found in order to improve the quality of life for HNSCC patients.

The circadian clock system is used by organisms to adjust their biochemical and behavioral processes to the cyclical environmental changes caused by the rotation of the Earth (12, 13). The master clock is located in the suprachiasmatic nucleus above the optic cross and is based on neural and humoral inputs that synchronize the cellular autonomous clock in peripheral organs with the “master clock” and thus regulate most life activities (14, 15). The circadian rhythm alteration in tumorigenesis may have the same importance as genetic factors and altered biological clock gene expression has been found in cancer cells (16–18). It has been shown that biological clock genes can directly or indirectly regulate the five major pathways of DNA repair and thus the progression of the cell cycle (19–21). There are also suggestions that dysregulation of circadian genes can contribute to inflammation and tumor progression through activation of p38, c-Myc, NF-kB, Bcl-XL, and protein kinase A (PKA) pathways (17, 22–25). Considering the significant role of CRRGs in tumors, several biological clock genes have been considered as prognostic biomarkers for various cancers (26–28). Currently, the prognostic value of CRRGs in HNSCC and the role of the tumor immune microenvironment are unclear. Hence, the purpose of this study was to develop a new risk-scoring system based on CRRGs to accurately predict prognosis and characterize the immune landscape of patients with CRRGs, providing a theoretical basis for personalized and precise clinical treatment.

With the continuous development of bioinformatics, a large quantity of articles was constructing prognostic models of diseases by filtering the prognostic markers of the features through methods as machine learning (29–31). In our study, we screened 6 reliable CRRGs by two machine learning methods, constructed a prognostic model based on the TCGA-HNSCC cohort, and went on to establish a risk score and comprehensively analyzed the relationship between CRRGs and immune microenvironment, immunotherapy, and chemotherapy sensitivity. We aimed to demonstrate the value of 6-CRRGs for assessing the prognosis of HNSCC patients through a comprehensive analysis of genomic data and to develop new tools to improve treatment options.

## 2 Method

### 2.1 Data sources

We downloaded gene expression profiles and clinical data of TCGA-HNSCC cohort including 504 tumor patients and 44 normal controls from the TCGA database (https://portal.gdc.cancer.gov/). The level 3 HTSeq-Fragments per kilobase million (FPKM) data of TCGA-HNSCC was converted to TPM (transcripts per million reads) according to the following formula: TPMn = FPKM_n_ * 10^6^/(FPKM_0_ +… + FPKM_m_), where n represented gene n and m represented the total number of all genes, respectively. Then, we performed a log_2_-based transformation of TPM. After excluding samples lacking complete clinical information, 501 HNSCC samples were included in the subsequent analysis. The sample size of HNSCC patients at the M stage varied greatly. This stage was consequently excluded from the analysis. We obtained 97 HNSCC patients with complete follow-up information from GSE41613 as an external validation set. In this study, two separate datasets, TCGA-HNSCC and GSE41613, were merged and the batch effect was corrected for using the ComBat function in the R package “sva” in order to eliminate the batch effect in the microarray expression data. We obtained Circadian rhythm-associated 1424 genes by Genecards (https://www.genecards.org/) (**Supplementary Table 1**).

### 2.2 Model construction

The “limma” R package was used to analyze CRRGs that are differentially expressed in HNSCC tissues and normal tissues. Univariate Cox regression analysis was performed to identify CRRGs with prognostic value. The prognostic network of DE-CRRGs associated with prognosis was mapped by the “igraph” R package. The obtained genes were further screened by Lasso and the RF algorithm, respectively. After the intersection of genes screened by the two methods, multivariate analysis was used for modeling and R package “glmnet” (32) was used to determine key genes and their regression coefficients. For each patient, the CRRGs risk score was calculated as follows, risk score = Expression_mRNA1_ × Coef_mRNA1_ + Expression_mRNA2_ × Coef_mRNA2_ +… Expression_mRNAn_ × Coef_mRNAn_.

### 2.3 Model validation

Subsequently, the HNSCC patients in the dataset were divided into low-risk and high-risk groups according to the median risk score. Overall survival (OS) was compared between the two groups using Kaplan-Meier curves created by survminer in the R package. The “pec” R package was used for PCA analysis and c-index calculation. The prediction accuracy of the signature was evaluated by constructing a time-dependent ROC using the “timeROC” R package.

### 2.4 Independent prognostic analysis and nomogram predictive model construction

Univariate Cox regression and multivariate Cox regression analysis were used to evaluate whether risk score was an independent prognostic factor. The rms R package combined with risk scores and clinicopathological characteristics was used to construct a nomogram for predicting the probability of survival at 1, 3, and 5 years in HNSCC patients.

### 2.5 Enrichment analysis

The “c2.cp.kegg.v7.4.symbols” obtained from the MSigDB database were analyzed using the “GSVA” R package. Gene Ontology (GO) analysis is done using the Cluster-Profiler R package.

### 2.6 Immunoassay of risk signatures

Currently recognized methods, including XCELL (33, 34), TIMER (35, 36), QUANTISEQ (37, 38), MCPCOUNT (39), EPIC (40), CIBERSORT (35, 41), and CIBERSORT-ABS (42)is used to measure immune infiltration correlation. The CIBERSORT algorithm was used to evaluate immune cell infiltration in HNSCC patients. Single sample GSEA (ssGSEA) was used to evaluate the immune function of the two groups. At the same time, we collected 19 inhibitory immune checkpoints with therapeutic potential from Auslander’s study (43). We obtained the gene set associated with cancer-immune circulation from the website developed by Xu et al. (http://biocc.hrbmu.edu.cn/TIP/) (44) and the gene set that was positively correlated with the clinical response to the anti-PD-L1 drug (atezolizumab) from the research features of Mariathasan (45). Using the GSVA algorithm (46) to calculate the enrichment scores of genetic signatures positively correlated with the cancer immune cycle and immunotherapy. Visualization was performed using the ggcor R software package. In addition, we analyzed the prediction of the 6-CRRGs signature on tumor immunotherapy response by the IMvigor210 cohort (http://research-pub.gene.com/IMvigor210CoreBiologies/).

### 2.7 Somatic mutation analysis

We downloaded the mutation data available to patients with TCGA-HNSCC from the TCGA Data Portal (https://portal.gdc.cancer.gov/). We analyze mutation data from HNSCC samples using maftools (47). The tumor mutation burden (TMB) score is calculated as follows: (Total Mutation/Total Coverage Base) × 10^^^6 (48). GSCALite (http://bioinfo.life.hust.edu.cn/web/GSCALite/) provides an online cancer genomic analysis platform by integrating 33 cancer data from TCGA and normal tissue genomics data from GTEx (49).

### 2.8 Database for tumor immune single-cells

A single-cell RNA sequencing database focused on the tumor microenvironment (TME) is housed at the Tumor Immunization Single Cell Center (TISCH)(http://tisch.comp-genomics.org/home/). Detailed cell type annotations are provided at the single cell level for further analysis of specific gene expression in different cell types. The specific gene expression in different cell types further reveals the variation of TME in patients with different HNSCC, thus explaining to some extent the heterogeneity of HNSCC.

### 2.9 Drug sensitivity

GDSC (Genomics of Drug Sensitivity in Cancer) (https://www.cancerrxgene.org/) was used to measure half-maximal inhibitory concentrations (IC50). This is achieved by using the pRRophetic R package.

### 2.10 Statistical analysis

Statistical analyses were performed using R software v4.1.3. Kaplan-Meier (KM) survival curves and log-rank tests were used to compare overall survival (OS) in the high-risk and low-risk groups. The LASSO regression analysis and RF were used to screen candidate CRRGs. Stepwise multifactor Cox regression analysis was used to construct CRRGs characteristics. Time-dependent ROC was used to assess the predictive performance of the model. Spearman correlation analysis was used to assess the correlation between risk score and immune cell infiltration. Wilcox test was used to compare the proportion of TIICs, immune checkpoints, and immune function between the two groups. *P* < 0.05 was considered statistically significant and false discovery rate (FDR) < 0.05 was considered statistically significant.

## 3 Results

### 3.1 Identification of candidate CRRGs

**Figure 1:**
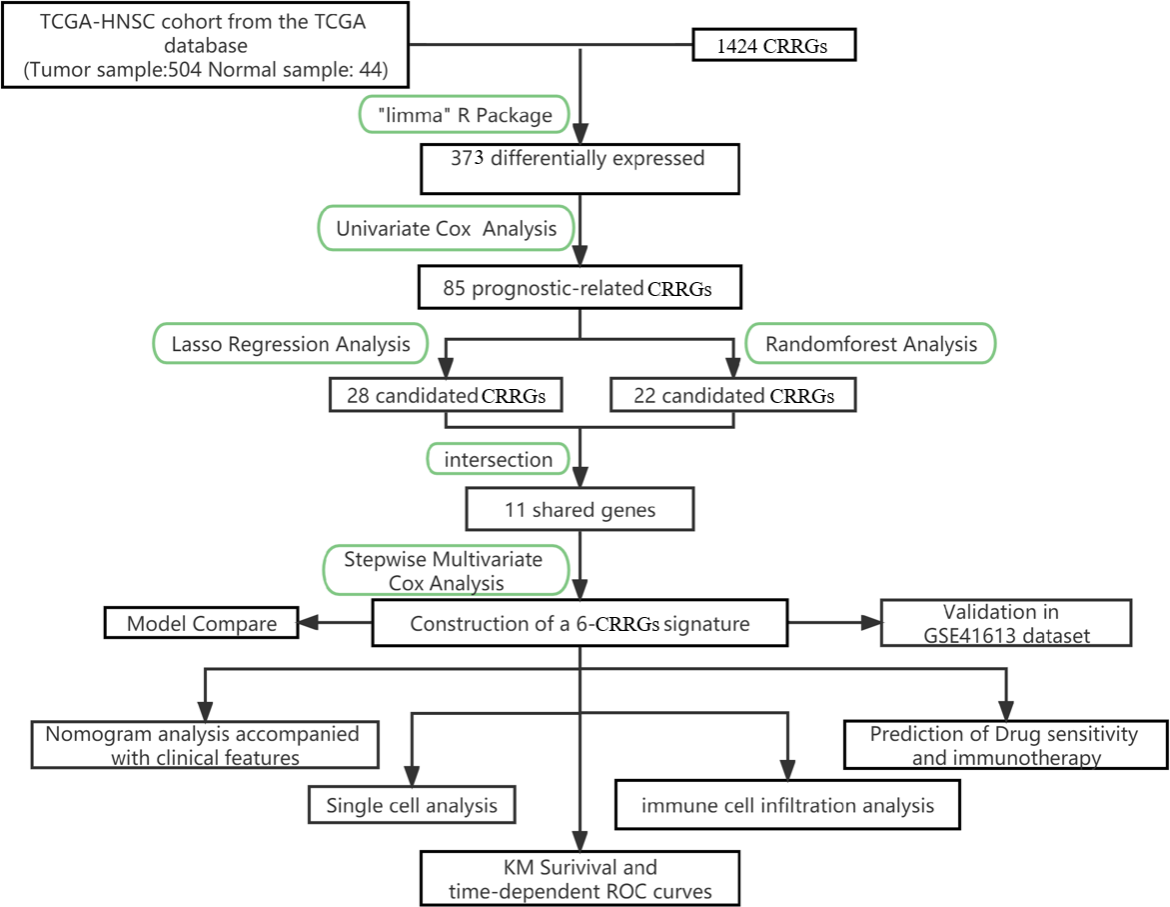
The flowchart summarizes the main design of the present study.

**Figure 2:**
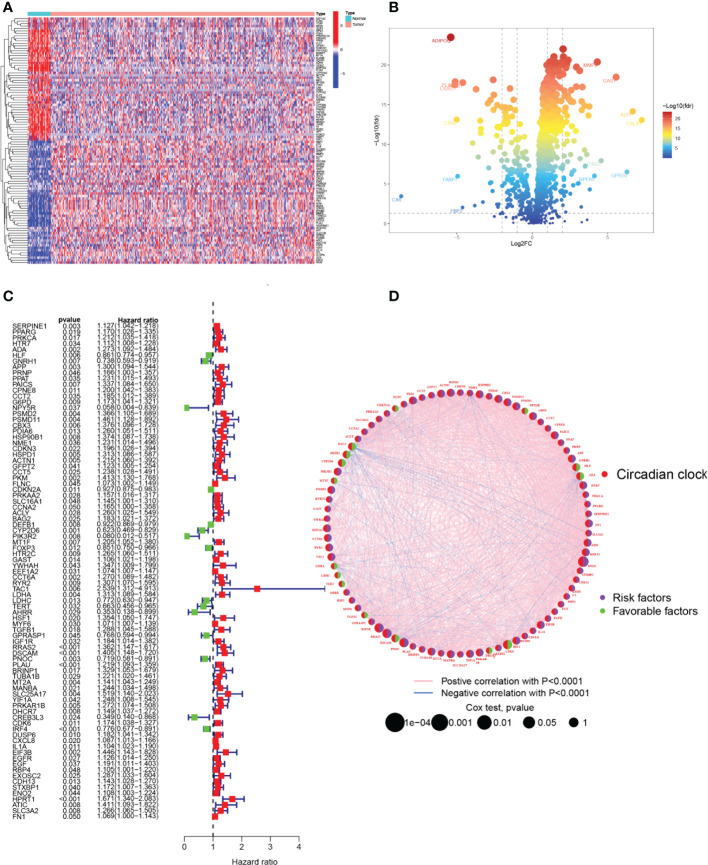
Identification of candidate CRRGs. **(A, B)** Heat map and volcano plot of differentially expressed CRRGs. **(C)** The prognosis of 85 CRRGs in the whole HNSCC cohort was analyzed by a univariate Cox regression model. **(D)** Correlation analysis of the expression of CRRGs after 87 COX regression analyses. The genes with favorable prognoses are marked in green and the harmful factors in purple. The size of the point indicates the *P*-value calculated by the COX regression, the larger the value, the smaller the point.

The primary design of this study can be known from the graphical flow chart (**Figure 1**). Aiming to explore CRRGs with differential expression and prognostic significance in HNSCC patients, we first performed differential expression analysis of gene expression in the normal and tumor groups by using the “limma” R package. After filtering the data according to thresholds (|log2FC|>1.0, FDR <0.05), we identified a total of 373 differential expressions (DE) CRRGs, of which 102 were down-regulated and 271 were up-regulated in HNSCC tissues (**Supplementary Table 2**). The DE-CRRGs were visualized by volcano plot and heat map (**Figures 2A, B**). The heatmap showed the top 50 CRRGs with the greatest upregulation of expression and the top 50 CRRGs with the greatest downregulation of expression relative to normal tissue. Univariate cox analysis yielded CRRGs that were associated with patients’ OS, and we ultimately identified 85 CRRGs that were prognostically significantly associated (**Figure 2C**). We then performed prognostic network mapping on them, and the CRRGs showed significant correlation and prognostic significance (**Figure 2D**). Based on these results, CRRGs may be associated with HNSCC tumorigenesis and progression.

### 3.2 Selection of modeling genes using LASSO and RF algorithms

**Figure 3:**
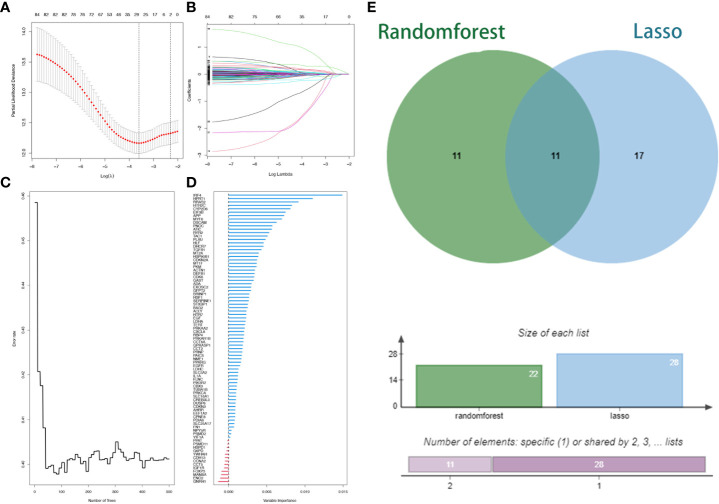
Lasso regression analysis and random survival forest screening for special issue genes. **(A)** Lasso coefficient curves. **(B)** Adjusted parameter selection in the Lasso model with tenfold cross validation. **(C, D)** RF error rate versus. **(E)** Wayne diagram showing intersection genes.

Two machine learning algorithms were applied to screen signature genes among biological clock genes associated with the prognosis of HNSCC patients. For the LASSO algorithm, cross-validation was performed. The minimum criteria for constructing the LASSO classifier were selected to identify 28 feature genes (**Figures 3A, B**). Combined with RF feature selection, the classification tree results (**Figures 3C, D**) were selected for genes with heavy variable importance > 0.004. Through crossover, a total of 11 feature genes including IL1B, TXN, and CASP3 were finally identified for LASSO, RF algorithm, and represented by the VENN diagram (**Figure 3E**). Subsequently, a stepwise multifactorial Cox risk regression algorithm was used to downscale these high-dimensional data, and the optimal Lambda value was 0.0273. Six prognosis-related CRRGs were finally identified for ADA, CYP2D6, RYR2, DSCAM, IRF4, and HPRT1. The corresponding regression coefficients were obtained as 0.2527, -0.6680, 0.3155, 0.3035, - 0.1897, and 0.5259. In multivariate Cox analysis, a linear prediction model was established based on the weighted regression coefficients of the six prognosis-related CRRGs, calculated as Risk score = (0.2527 × ADA expression level) + (-0.6680 × CYP2D6 expression level) + (0.3155 × RYR2 expression level) + (0.3035 × DSCAM expression level) + (-0.1897 × IRF4 expression level) + (0.5259 × HPRT1 expression level).

### 3.3 Validation of 6-CRRGs signature

**Figure 4:**
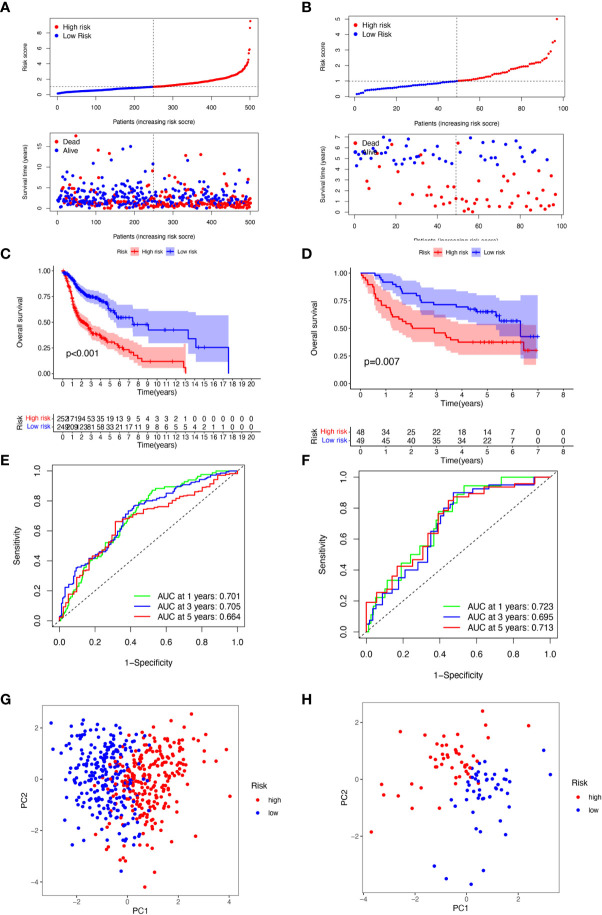
Validation of 6-CRRGs Signature. **(A, B)** Distribution of risk scores and patient survival between low and high-risk groups in the TCGA cohort and the GEO cohort. **(C, D)** KM curve compares the overall HNSCC patients between low- and high-risk groups in the TCGA cohort and the GEO cohort. **(E, F)** Time-dependent ROC curves analysis in the TCGA cohort and the GEO cohort. **(G, H)** PCA plot in the TCGA cohort and the GEO cohort.

For the purpose of evaluating the stability of the constructed risk model in predicting patient prognosis, we used the TCGA-HNSCC cohort as the internal training and the GSE41613 cohort as the external validation cohort. Risk scores were calculated separately for each sample in the TCGA training and validation cohorts based on the same risk formula, and we could find that when the risk of HNSCC patients was elevated in both cohorts, patients showed a survival disadvantage of reduced OS and increased mortality (**Figures 4A, B**). Based on the median risk score, we were able to divide the patients into two subgroups of high and low risk to explore prognostic differences. The Kaplan-Meier curves showed a significant difference in prognosis between the high and low-risk patients in these two cohorts respectively, with patients in the low-risk group having a more significant survival advantage (**Figures 4C, D**). The ROC curve (Receiver operating characteristic curve) was used as a tool to predict the survival time of patients at 1, 3, and 5 years. The AUC values for the TCGA-HNSCC cohort were 0.701, 0.705, and 0.664 respectively while the AUC values for the GSE41613 cohort were 0.723, 0.695, and 0.713 respectively (**Figures 4E, F**). This indicates that the model has an excellent predictive effect. Based on a principal components analysis of the risk model, both high-risk and low-risk patients in the two cohorts showed significant differences and were successfully divided into two relatively independent clusters (**Figures 4G, H**). These results confirm that the risk model has stable and excellent generalisability and predictive capability.

### 3.4 Clinical correlation and survival analysis of 6-CRRGs in patients with HNSCC

**Figure 5:**
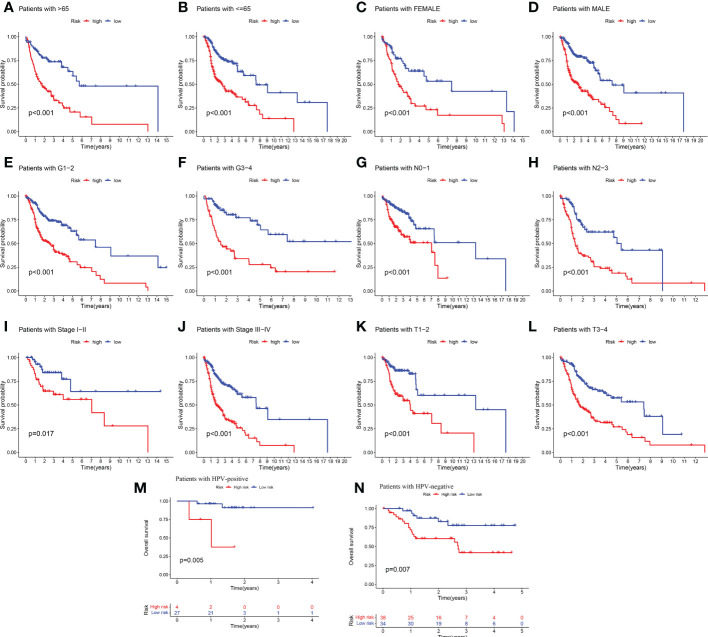
Clinical correlation and survival analysis of 6-CRRGs in patients with HNSCC **(A, B)** age, **(C, D)** gender, **(E, F)** tumor grade, **(G, H)** N stage, **(I, J)** tumor stage, **(K, L)** T stage, and **(M, N)** HPV status.

Given the significant differences in OS between high and low-risk groups in individual clinical characteristics, in order to explore and compare such differences in a more focused manner, we divided HNSCC patients into six different subgroups based on clinical characteristics. Namely, pathological stage (I-II and III-IV), Age (≤65 and >65 years), gender (female and male), pathological grading (G1-2 and G3-4), N-stage (N0-1 and N2-3), T-stage (T1-2 and T3-4) and HPV status (positive and negative). Notably, in all subgroups, low-risk patients had a significant survival advantage of longer survival time compared to high-risk patients (**Figure 5**). Based on the analysis of the results, we are more confident that the 6-CRRGs risk model is a reliable clinical prediction tool.

### 3.5 Correlation analysis of risk scores with clinical characteristics

To provide an analysis of the association between high and low-risk groups and clinical characteristics, a heatmap was made based on clinical characteristics, risk, and gene expression, which showed the correlation between CRRGs established in the prognostic risk model and the clinical characteristics and risk scores of all HNSCC patients in TCGA as a whole group (**Supplementary Figure 1A**). By comparing the distribution of clinical characteristics between the high and low-risk groups, we found significant differences in the distribution of clinical stage, grading, T-stage, and N-stage, while age and gender did not vary significantly between the two subgroups (**Supplementary Figure 1B**). The Wilcoxon test was then used to compare the differences in risk scores for clinical characteristics between the subgroups to test the correlation between both. Risk scores were found to be significantly associated with grade (*P*<0.01), T stage (*P*<0.01), stage (*P*<0.01), and N stage (*P*<0.05), but not with age, or gender (**Supplementary Figures 1C–H**).

### 3.6 Creation of nomograms based on 6-CRRGs signatures combined with clinical characteristics

**Figure 6:**
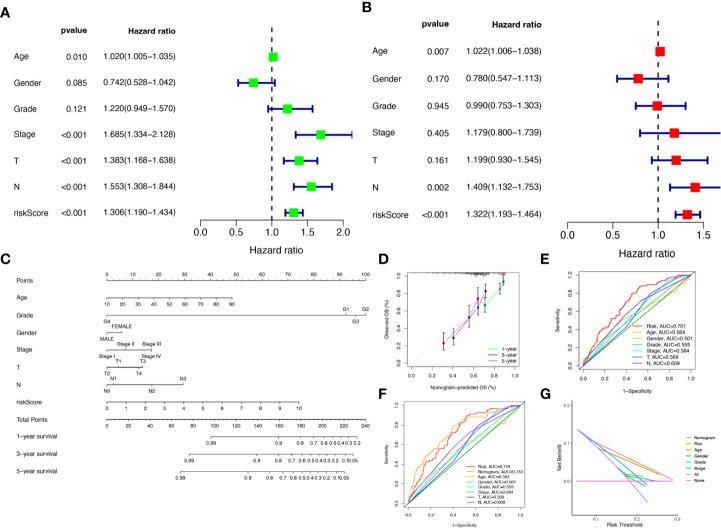
Creation of nomograms based on 6-CRRGs signatures combined with clinical characteristics. **(A)** Univariate and **(B)** multivariate COX regression analysis of the signature and different clinical features. **(C)** A nomogram combining risk score, age, grade, gender, stage, T stage, and N stage. **(D)** The calibration curve of the constructed nomogram of 1-year, 3-year, and 5-year survival. **(E)** Time-dependent ROC curves analysis. **(F)** The nomogram’s time-dependent ROC curves. **(G)** Decision curve analysis.

To verify the credibility and clinical value of the biological signature constructed based on the 6-CRRGs as a predictor of prognosis, we included each HNSCC patient’s risk score with common clinical indicators for comparison and observed the correlation between each factor and patient prognosis after successive univariate Cox and multivariate Cox analyses. Based on the analysis of the results, it is clear that Stage, T-stage, N-stage, and risk score (*P*<0.001) were all prognostic factors significantly associated with patient prognosis in the univariate cox analysis (**Figure 6A**). However, after multifactorial cox analysis, only the risk score (*P*<0.001) and N stage (*P*=0.002) remained significant (**Figure 6B**). We further compared risk, N stage, and the remaining clinical indicators and it is worth noting that risk (AUC=0.701) was superior to N stage (AUC=0.609) and the other indicators (**Figure 6E**). It suggests that the risk score is the most reliable factor as an independent predictor of patient prognosis. Based on the above analysis, the hope of being able to predict patients’ prognoses quantitatively and inform clinical decision-making. We integrated the risk score and its clinical indicators to construct a Nomogram plot as a means of predicting the probability of prognostic survival at 1, 3, and 5 years (**Figure 6C**). Calibration analysis showed that the prediction curves for OS for patients at 1, 3, and 5 years were highly similar to the ideal 45-degree calibration line, indicating excellent stability of the Nomogram plot (**Figure 6D**). We then compared Nomogram, risk, and common clinicopathological features, with risk (AUC=0.718) and Nomogram (AUC=0.743) having more accurate predictive performance and discriminatory power than a single independent clinical indicator (**Figure 6F**). Subsequently, DCA (Decision curve analysis) showed that Nomogram and risk yielded greater net benefit and predictive benefit, indicating that both the model’s risk score and nomogram could be used as primary decision factors (**Figure 6G**). Combined with the above results, this suggests that our 6-CRRGs risk model is more practical and influential for clinical decision-making and is more suitable as a clinical decision tool for predicting the prognosis of HNSCC patients in the clinical setting.

### 3.7 Functional enrichment analysis of CRRGs in HNSCC

**Figure 7:**
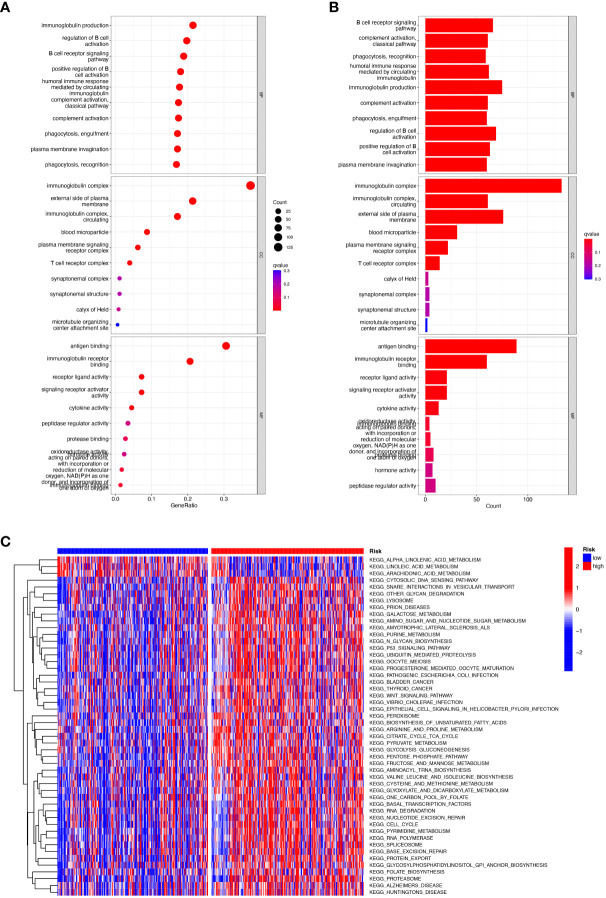
Functional enrichment analysis of CRRGs in TCGA-HNSCC. **(A, B)** Gene Ontology (GO) enrichment analysis was used to analyze the differential genes between HNSCC and normal samples. **(C)** GSVA analysis between the high-risk cohort and the low-risk cohort.

We performed gene ontology (GO) analysis and Kyoto Encyclopedia of Genes and Genomes (KEGG) analysis on DEGs to elucidate the information pathways and underlying molecular mechanisms of the risk signature. After filtering the data according to thresholds (|log2FC|>1.0, FDR<0.05), we could obtain enrichment results that were significantly different in the HNSCC and normal groups. Cellular fractions (CC) mainly include immunoglobulin complex, external side of plasma membrane and immunoglobulin complex, circulating, etc. Molecular functions (MF) include mainly antigen binding and immunoglobulin receptor binding. Biological processes (BP) include immunoglobulin production, regulation of B cell activation, B cell receptor signaling pathway and humoral immune response, etc. (**Figures 7A, B**). KEGG and GSVA further explored enrichment pathways that differed significantly between high and low-risk groups, ultimately identifying 91 significantly enriched pathways (**Figure 7C**), including other relevant KEGG pathways such as base excision repair, and protein export in the high-risk group. The low-risk group included KEGG pathways related to α-linolenic acid metabolism, metabolism of linoleic acid, and metabolism of arachidonic acid. It is worth noting that the results of the GO enrichment analysis were mainly related to immune-related functions and biological processes, so we performed a more comprehensive and detailed analysis of the immune landscape of patients in the high and low-risk groups.

### 3.8 Multi-omics mutation analysis of 6-CRRGs

**Figure 8:**
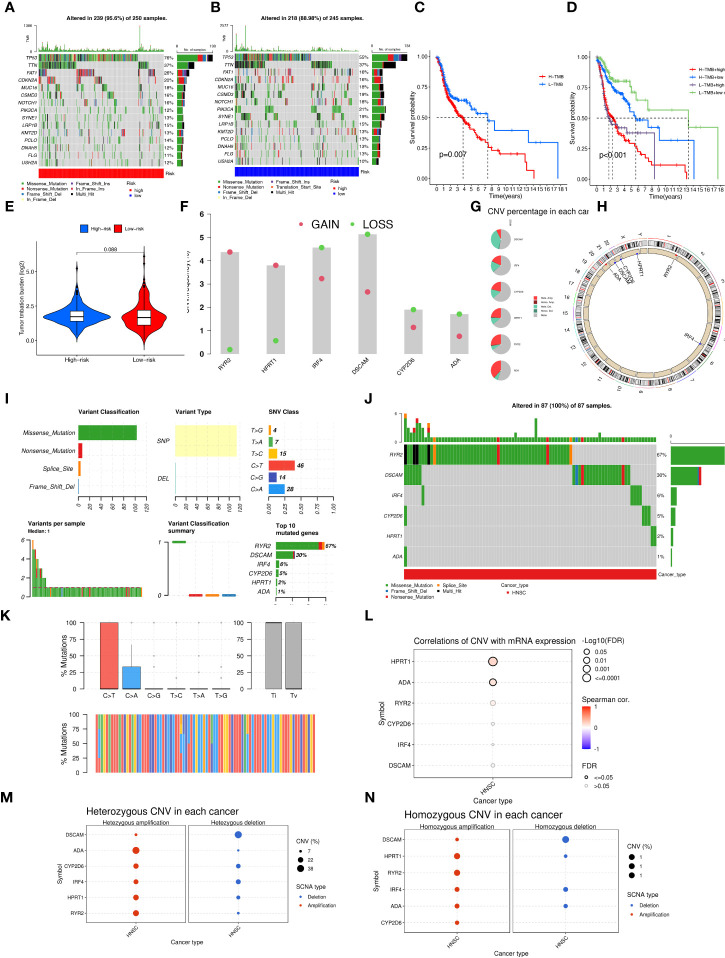
Multi-omics mutation analysis of 6-CRRGs. **(A)** Somatic mutations in the high-risk group. **(B)** Somatic mutations in the low risk group. **(C)** The KM curve compares the overall HNSCC patients between high and low mutation groups. **(D)** KM curve based on mutation and risk scores for the four subgroups compares the overall HNSCC patients between. **(E)** TMB analysis between high- and low-risk groups. **(F)** Copy number trends of 6-CRRGs. **(G)** CNV occupancy. **(H)** Copy number change circle plot. **(I)** Mutations of 6-CRRGs in TCGA-HNSCC patients. **(J)** Mutation analysis of 6-CRRGs in somatic cells. **(K)** SNV analysis. **(L)** Copy number variation and gene expression Spearman correlation coefficient analysis. **(M)** Heterozygous mutations. **(N)** Homozygous mutations.

Aberrant mutations and copy number variants in somatic cells may have potential relevance in tumourigenesis and progression. We first visualized the distribution of mutated genes between high and low-risk groups with the ''maftools'' R package to see if the combined landscape of mutation profiles differed between the two subgroups. Through the results, we noted the distribution of the top 15 mutated genes with the highest frequency of change, with a higher frequency of somatic mutations in the high-risk group (95.6%) than in the low-risk group (88.98%) (**Figures 8A, B**). with the highest mutation frequencies being TP53, TTN, and FAT1 in that order. The analysis revealed significant differences in prognosis between the high and low TMB groups. Based on these results, we collaborated on the effects of TMB and risk scores for prognostic analysis, and we obtained four subgroups and found that patients with low TMB and low risk had a much better prognosis than the other subgroups (**Figures 8C, D**). However, there was no significant difference between the TMB and high and low-risk groups (**Figure 8E**). In addition, we explored the incidence of CNV mutations in the risk signature, where RYR2 and HPRT1 copy numbers tended to be amplified, while IRF4, DSCAM, CYP2D6, and ADA tended to be absent (**Figure 8F**). **Figure 8G** showed the percentage of CNV in HNSCC for 6-CRRGs. The mutational sites and mutational trends of 6-CRRGs on chromosomes are shown in **Figure 8H**. To clarify the biological mechanisms underlying the aberrant expression of 6-CRRGs in HNSCC patients, we investigated the single nucleotide locus variants (SNVs) of 6-CRRGs in patient tissues. Analysis of the results showed that missense mutations are most likely to occur in HNSCC patients and that single nucleotide polymorphisms (SNPs) are a common variant type (**Figure 8I**). Risk signatures in somatic mutations were present in a total of 87 HNSCC patients, with the highest frequency of mutations being in RYR2 (**Figure 8J**). SNV Classes were mainly C>T and C>A (**Figure 8K**). In addition, Spearman's correlation coefficient analysis of copy number variants and gene expression revealed that HPRT1 and ADA showed upregulated copy number variants (**Figure 8L**). We explored heterozygous and homozygous mutations and showed that the heterozygous copy number amplification was mainly in ADA and RYR2, while DSCAM was mainly realized as deletion. Homozygous mutations were mainly HPRT1 and RYR2 amplifications, while DSCAM again showed a reduction in copy number, so the abnormal gene expression could be the result of both copy number variation and single nucleotide variation (**Figures 8M, N**).

### 3.9 6-CRRGs signature predicts TME and immune cell infiltration

**Figure 9:**
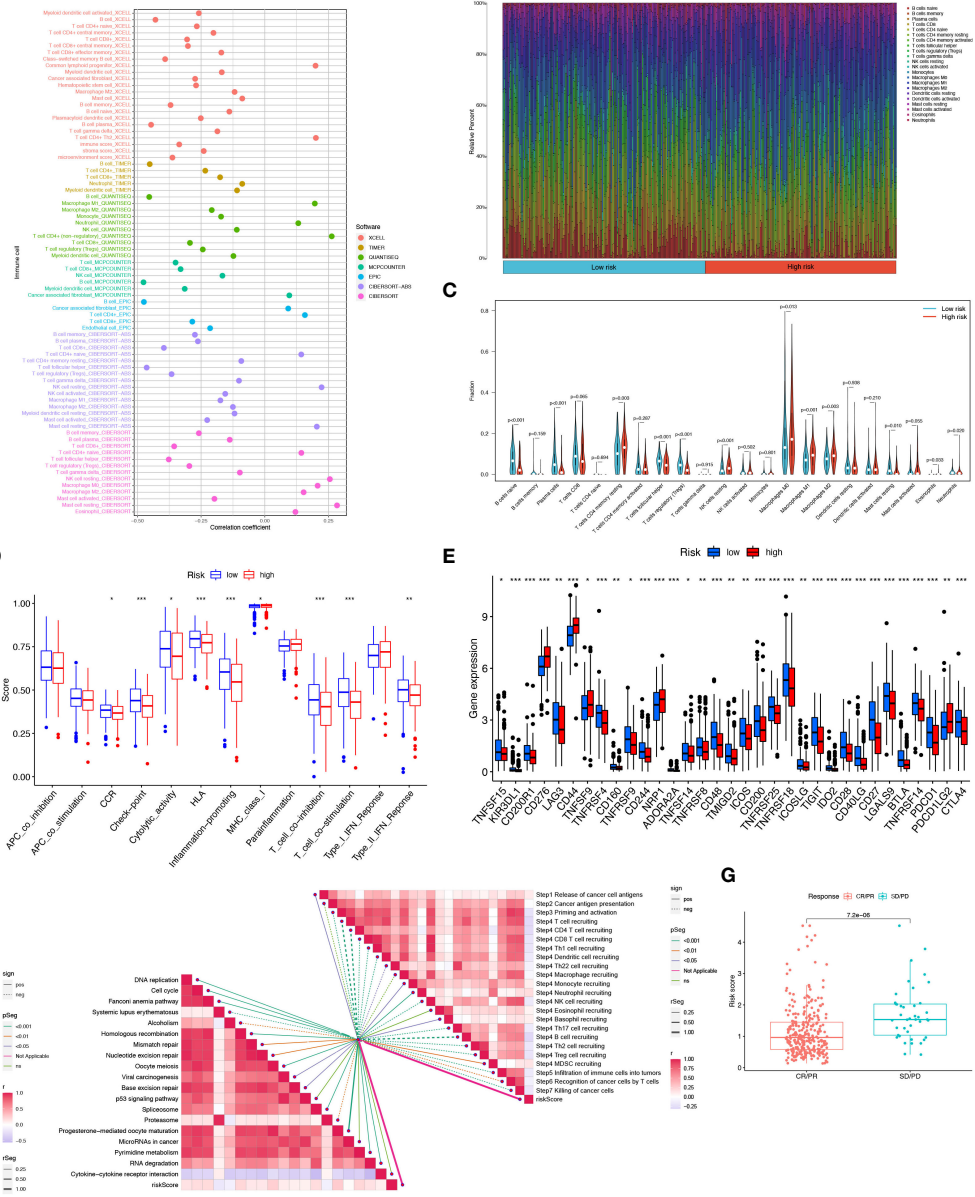
6-CRRGs risk score predicts TME and immune cell infiltration. **(A)** Immune cell bubble map. **(B)** Barplot showing the proportion of 22 kinds of TICs in HNSCC tumor samples. **(C)** Differences in immune cell infiltration between high- and low-risk groups. **(D)** Immune cell and immune function ssGSEA scores between high- and low-risk groups. **(E)** Immune checkpoint differences between high- and low-risk groups. **(F)** Correlation between risk score and ICB response signature. And the correlation of risk scores with each step of the tumor immune cycle. * *P <*0.05; ** *P <*0.01; *** *P <*0.001 **(G)** Correlation between risk scores and clinical response to cancer immunotherapy.

Crosstalk between cancer cells and TME is closely related to tumor proliferation, invasion, and metastasis [27]. And while Tumor-infiltrating immune cells (TIICs) are one of the important components of TME, their composition, and distribution are inextricably linked to the process of tumor development. First, we explored the correlation between risk score and infiltrating immune cell abundance based on a total of seven different algorithms, EPIC, XCELL, CIBERSORT, MCPCOUNTER, QUANTISEQ, CIBERSORT-ABS and TIMER, where CD4+ T cell infiltration was positively correlated with risk score in most algorithms (**Figure 9A**). To bring the distribution of immune infiltration in HNSCC into full view to further explore the specific immune phenotype of HNSCC. Next, we explored the immune infiltration of 22 immune cell subpopulations inferred from mRNA expression in HNSCC patient tissues in the TCGA database by the CIBERSORT algorithm and investigated the differences between the high- and low-risk groups. The levels of the different types of immune cell populations in each HNSCC patient sample can be obtained by looking at the length of the various colors in the bar graph. From the graphs, we found a relatively high proportion of M0, M1, and M2 macrophages and T cells CD8, T cells CD4 memory resting in the tissues of HNSCC patients, accounting for approximately 57% of the 22 immune cell subpopulations. Instead, the percentage of eosinophils, Monocytes, and Neutrophils were relatively low at approximately 10% (**Figure 9B**). Meanwhile, since the composition of the tumor immune microenvironment significantly affects tumor growth, some studies have confirmed whether circadian rhythm disorders affect the ratio of various immune cells in the tumor microenvironment and ultimately promote tumor progression. The expression of Neutrophils, Eosinophils, Macrophages M2, Macrophages M1, and T cells CD4 memory resting was significantly higher in the high-risk group (**Figure 9C**). As immune cells with immune checkpoints can significantly influence immune function, we compared ssGSEA scores for immune function and several immune function scores were significantly higher in the low-risk group than in the high-risk group (**Figure 9D**). Owing to the importance of checkpoint-based immunotherapy, we analyzed the expression of immune checkpoint genes in the high-risk and low-risk groups. Most immune checkpoint genes were found to be significantly upregulated in the low-risk group, including IDO2, CTLA-4, TIGIT, KIR3DL1, PDCD1, and CD28 (**Figure 9E**), indicating that patients in the low-risk group may have better efficacy with ICB treatment. As ICB response plays an important role in immune checkpoint therapy, we further analyzed the correlation between risk score and ICB response signature and found that of these, only Systemic lupus erythematosus, Alcoholism, and Proteasome showed a significant negative correlation with a risk score. No significant correlations were found with the p53 signaling pathway, MicroRNAs in cancer, and Cytokine-cytokine receptor interaction with risk scores. In contrast, the other immune cycle steps were positively correlated with our risk score. A correlation analysis between risk score and ICB response signature was also performed, in which we found that the Re-lease of cancer cells, Monocyte recruiting, Neutrophil recruiting, Basophil recruiting, and MDSC recruiting (step 4) interactions were significantly positively correlated, only Cancer antigen presentation, Macrophage recruiting and Eosinophil recruiting (step 4) were not significantly correlated with risk scores, while the other immune cycle steps were negatively correlated with our risk scores (**Figure 9F**). More importantly, the expression of 6-CRRGs was significantly higher in patients with progressive and stable disease than in those in partial or complete remission (*P*=7.2e-06) (**Figure 9G**). To examine the potential of risk scores in predicting immunotherapy from a real immunotherapy cohort, we selected IMvigor210 as the cohort of patients receiving immunotherapy. The distribution of immune response outcomes in the high- and low-risk groups is shown in **Supplementary Figure 2A**, which shows that low-risk patients are more likely to produce an immune response, possibly predicting that low-risk patients can produce better therapy when receiving immunotherapy (**Supplementary Figure 2B**). Where the 1, 3, and 5-year predictive sensitivity was tested by ROC curves with satisfactory AUC values (**Supplementary Figure 2C**). The survival curves of patients in the high and low risk groups of the cohort clock were remarkably different, with a meaningful survival advantage for low risk patients (**Supplementary Figure 2D**).

### 3.10 6-CRRGs signatures have better prognostic predictive performance than other signatures

For further demonstration of whether our constructed 6-CRRGs signature has an accurate predictive capability for HNSCC patients, we collected five published prognostic signatures, namely Liu signature (50), Jiang signature (51), Chen signature (52), Huang signature (53) and Wang signature (54) (**Supplementary Figures 3A–F**). To increase the comparability of signatures and to ensure fairness of comparison, we obtained risk coefficients for each gene using the same modeling approach, from which we were able to calculate the risk score for each HNSCC sample in the entire TCGA cohort. Furthermore, using the median value of the risk scores for all samples allowed the samples to be divided into two prognostic subgroups of high and low risk. It was found that while all five signatures, except the Chen signature, were effective in classifying HNSCC patients into two subgroups with significantly different prognoses, its ROC curves were not satisfactory in predicting AUC values for 1-, 3- and 5-year survival, all being significantly lower than the AUC values of our model. More notably on the C-index analysis, it also showed that our signature performed remarkably better than the other signatures (**Supplementary Figure 3G**). The above results suggest that our constructed signature of 6-CRRGs exhibits more accurate and stable predictive performance than the other signatures.

### 3.11 Correlation analysis of immune microenvironment and 6-CRRGs signature

Based on the single-cell dataset of HNSCC_GSE103322 in the TISCH database, we analyzed the correlation between the expression of 6-CRRGs and the immune microenvironment. There are 20 cell populations and 11 immune cell types stored in the GSE103322 dataset (**Supplementary Figures 4A, B**), and the distribution and numbers of various cell types are clearly visible (**Supplementary Figure 4C**). The expression levels of 6-CRRGs in various immune cells were observed in **Supplementary Figures 4D–I**, where ADA and HPRT1 were expressed on various immune cells, while IRF4 was mainly expressed in CD4Tconv, CD8T, and CD8Tex. Unfortunately, we found that CYP2D6, RYP2H, and DSCAM were barely expressed in the immune microenvironment.

### 3.12 6-CRRGs signature predicts chemotherapy sensitivity

**Figure 10:**
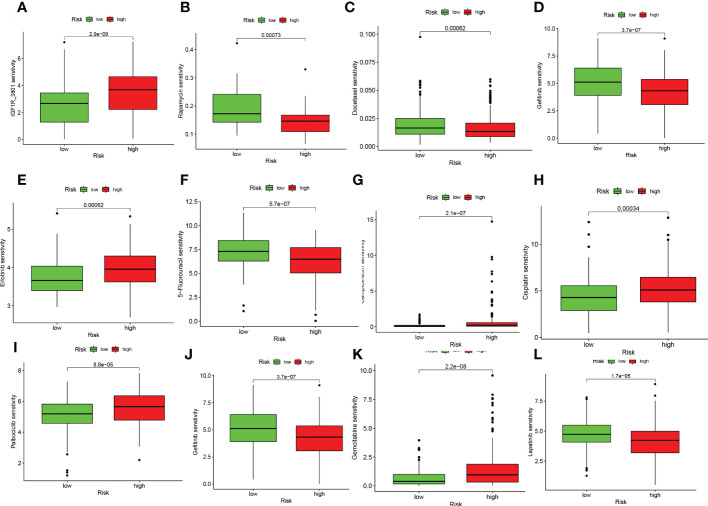
6-CRRGs signature predicts chemotherapy sensitivity. **(A)** IGF1R_3801, **(B)** Rapamycin, **(C)** Docetaxel, **(D)** Paclitaxel, **(E)** Erlotinib, **(F)** 5-Fluorouracil, **(G)** Camptonthecin, **(H)** Cisplatin, **(I)** Palbociclib, **(J)** Gefitinib, **(K)** Gemcitabine, **(L)** Lapatinib.

On the basis of risk scores, to evaluate the potential biological value of 6-CRRGs as biomarkers for predicting response to clinical drug therapy in patients with HNSCC, we analyzed the IC50 values of 198 drugs between high and low risk groups of patients with TCGA-HNSCC by the “pRRophetic” R package. We found 142 chemotherapeutic or targeted agents with significantly different chemosensitivity. In **Figure 10** we showed that in the low-risk group Rapamycin (*P*=0.00073), Docetaxel (*P*=0.00062), Gefitinib (*P*=3.7e-07), 5-Fluorouracil (*P*=5.7e-07), Lapatinib (*P*=1.7e-05) and Paclitaxel (*P*=1.1e-10) possessed higher IC50 values. In contrast, among the remaining six chemical or targeted agents IGF1R_3801 (*P*=2.9e-09), Erlotinib (*P*=0.00062), Camptothecin (*P*=2.1e-07), Cisplatin (*P*=0.00034), Palbociclib (*P*=8.8e-05) Gemcitabine (*P*=2.2e-08) had higher IC50 values at higher risk.

## 4 Discussion

Despite the heavy health burden of HNSCC on society, circadian rhythm-associated genes have lacked systematic studies in HNSCC. Therefore, we constructed a multigene prognostic model based on circadian rhythm-related genes based on the TCGA-HNSCC dataset. In this study, we selected 6-CRRGs to construct a new prognostic model using Lasso, RF, and univariate and multifactor COX risk regression analyses. The signature of the 6-CRRGs we constructed proved to be an independent prognostic factor for HNSCC. We found significant prognostic differences between the two groups. ROC curve and calibration curve analyses demonstrated the outstanding predictive performance of the 6-CRRGs signature. In addition, we constructed a nomogram and found that the 6-CRRGs signature has better predictive efficacy compared to clinicopathological features, which can help clinicians to make more accurate prognostic judgments for HNSCC patients.

It is now well established that adenosine is able to bind to A2AR and thereby limit the antitumor activity of CD4+ T cells, CD8+ T cells, and NK cells, and that it induces the expression of CTLA-4 and PD-1 to promote immune escape (55, 56). ADA is a key enzyme that protects T cells from adenosine inhibition, and its absence would promote tumor progression (57, 58). On this basis, ADA activity was considered an indicator of immune function in patients with HNSCC (59, 60). Cytochrome P450 2D6 (CYP2D6) is a catabolic enzyme of some commonly used drugs. Individuals with the CYP2D6PM genotype have been reported to be more susceptible to HNSCC and to have a greater impact on treatment response (61). mutations in ryanodine receptor 2 (RYR2) are commonly thought to be strongly associated with lethal arrhythmias and heart failure (62, 63). and subsequent studies have shown that RYR2 somatic mutations and promoter methylation were shown to contribute to the pathogenesis of HNSCC (64). Hypoxanthine phosphoribosyl transferase 1 (HPRT1) regulates the production of purines and inosine involved in the cell cycle and can enhance chemoresistance *via* the MMP1/PI3K/Akt axis in patients with oral squamous cell carcinoma (65).

TME has become a consensus as a key factor influencing the development of cancer, and the immune microenvironment should be noted as one of the main features of TME. Treg cells, as key cells regulating immune responses and maintaining self-tolerance, cause CD8+ T cell dysfunction in TME in HNSCC (66–68). In addition, despite the higher degree of infiltration of M1-type macrophages in the high-risk group, it has been shown that M2 is the predominant macrophage type in TME of HNSCC and is able to suppress the antitumor effects of M1 by secreting various cytokines (69–71). Further integrating the results of immune cell, and immune function analysis, we hypothesize that both low- and high-risk groups of HNSCC patients may have a suppressive immune microenvironment and limited immune cell infiltration different from other common malignancies. Given the immunosuppression and therapeutic resistance caused by complex TME, immunotherapy has great potential for oncology treatment (72). Ipilimumab is an FDA-approved CTLA-4 inhibitor and has been tested in various clinical trials in patients with HNSCC (NCT02369874, NCT02551159, NCT02319044) (73–76). One case reported a satisfactory outcome with nabumab in combination with ipilimumab in a patient with refractory HNSCC (77). Unfortunately, although immune checkpoint blockade (ICB)-based immunotherapies have some potential for HNSCC, most patients have satisfactory efficacy against them, and the intensity of the stimulatory co-signal hardly exceeds that of the heavy inhibitory factor in TME (78, 79). Therefore, it is crucial to screen patients sensitive to various immune checkpoint therapies based on their expression of immune checkpoint genes, and our model has promising results in this regard.

High tumor mutational load is usually considered to be strongly associated with a good prognosis due to increased immunogenicity of tumor-specific antigenic targets and tumor-infiltrating immune cells (80). However, the opposite result has been observed in HNSCC, possibly due to the low immunogenicity of HNSCC (81). The search for new antigens that are widely mutated in HNSCC is crucial for targeted therapy. TP53, due to its ability to halt tumor progression by regulating apoptosis, angiogenesis, and DNA repair, may promote tumor cell metastasis through the accumulation of p53 molecules compared to wild-type TP53 leading to poor prognosis in HNSCC patients (82, 83). FAT1 has two opposing mechanisms, on the one hand, binding ß-catenin to prevent tumor progression. On the other hand, interaction with Ena/VAPS and Scribble promotes cell invasiveness and metastasis, which requires additional experimental studies under different conditions (84–86). These highly mutated genes in HNSCC provide new directions and perspectives for subsequent targeted therapies, which may lead to better benefits for patients.

Although the 6-CRRGs signature we constructed has promising value in predicting the prognosis of HNSCC patients and helping clinicians with treatment selection, we still need to acknowledge that this study has some limitations. Firstly, our study was based on the analysis of data from public databases, which may lead to deviations in predictions from the actual situation, although we have taken several approaches to try to avoid this. There is still a need to collect a large amount of clinical information on HNSCC patients in the clinic as well as sequencing data after receiving immunotherapy in order to validate the accuracy of the practical application of the model and the accuracy of predicting response to immunotherapy and chemotherapy. Finally, we did not consider tumor location in this study because HNSCC contains too many sites of incidence, and the number of patients varies too much from site to site. In TCGA, some of the tumors had only single-digit sample sizes and could not be studied. This is, of course, one of the limitations of our study.

## 5 Conclusion

In summary, we systematically explored 6-CRRGs signature for HNSCC and successfully constructed a prognostic signature of 6-CRRGs, which can accurately assess the prognosis and immune status of HNSCC patients and help clinicians identify specific sub-groups of patients who may benefit from immunotherapy and chemotherapy for personalized treatment.

## Data availability statement

The original contributions presented in the study are included in the article/**Supplementary Material**. Further inquiries can be directed to the corresponding authors.

## Author contributions

GT and ZX conceived the study. HC, JY, and GP drafted the manuscript. HC, JZ, and XX performed the literature search and collected the data. GS, XX, and JZ analyzed and visualized the data. JL, ZX, and GT helped with the final revision of this manuscript. All authors contributed to the article and approved the submitted version.

## Supplementary material

Supplementary Table 1 1424 CRRGs were obtained through Genecards database.

Supplementary Table 2 Differential expression analysis of CRRGs in HNSCC and normal tissues.
