# Supplementary material for: Circadian rhythm-related genes index: A predictor for HNSCC prognosis, immunotherapy efficacy, and chemosensitivity
Source: Front Immunol. 2023 Mar 10;14:1091218. doi: 10.3389/fimmu.2023.1091218 (PMC10036372; doi:10.3389/fimmu.2023.1091218)
Supplement: Supplementary file 3 [file DataSheet_1.docx]

Supplementary Material

## Supplementary Figures


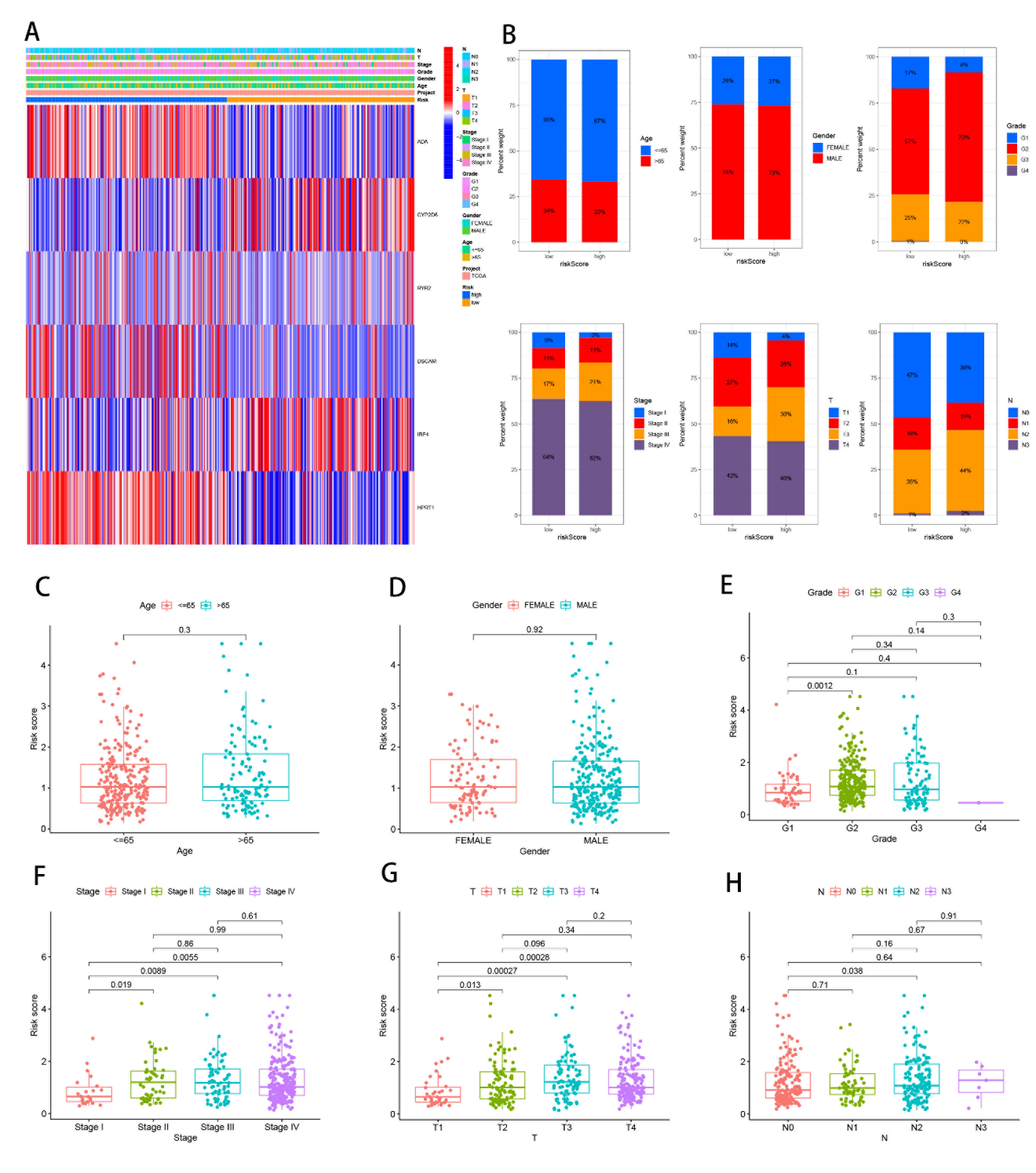


**Supplementary Figure 1** Correlation analysis of risk scores and clinicopathological characteristics. (A) Heatmap for the 6-CRRGs based signature with clinicopathological manifestations. (B) Risk score distribution is stratified by age, gender, grade, tumor stage, T stage, and N stage. The distribution of risk scores according to (C) age, (D) gender, (E) grade, (F) tumor stage, (G) T stage, and (H) N stage.


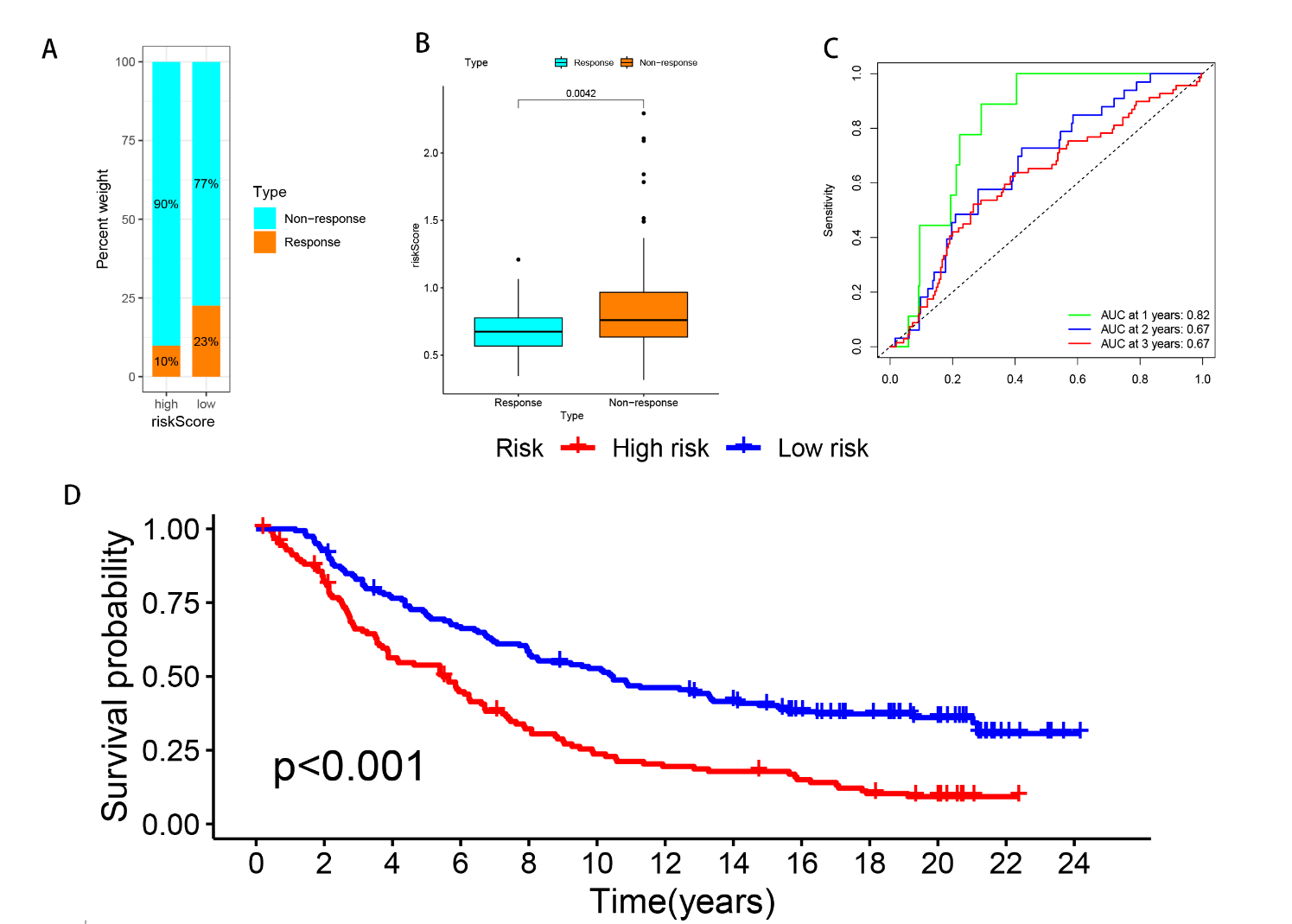


**Supplementary Figure 2** Immunotherapy cohort effect based on 6-CRRGs (A) Stacked plot of immune response distribution in high and low risk groups. (B) Box-line plots of immune response profiles in high- and low-risk groups. (C) ROC curves. (D) KM curves between high- and low-risk groups.


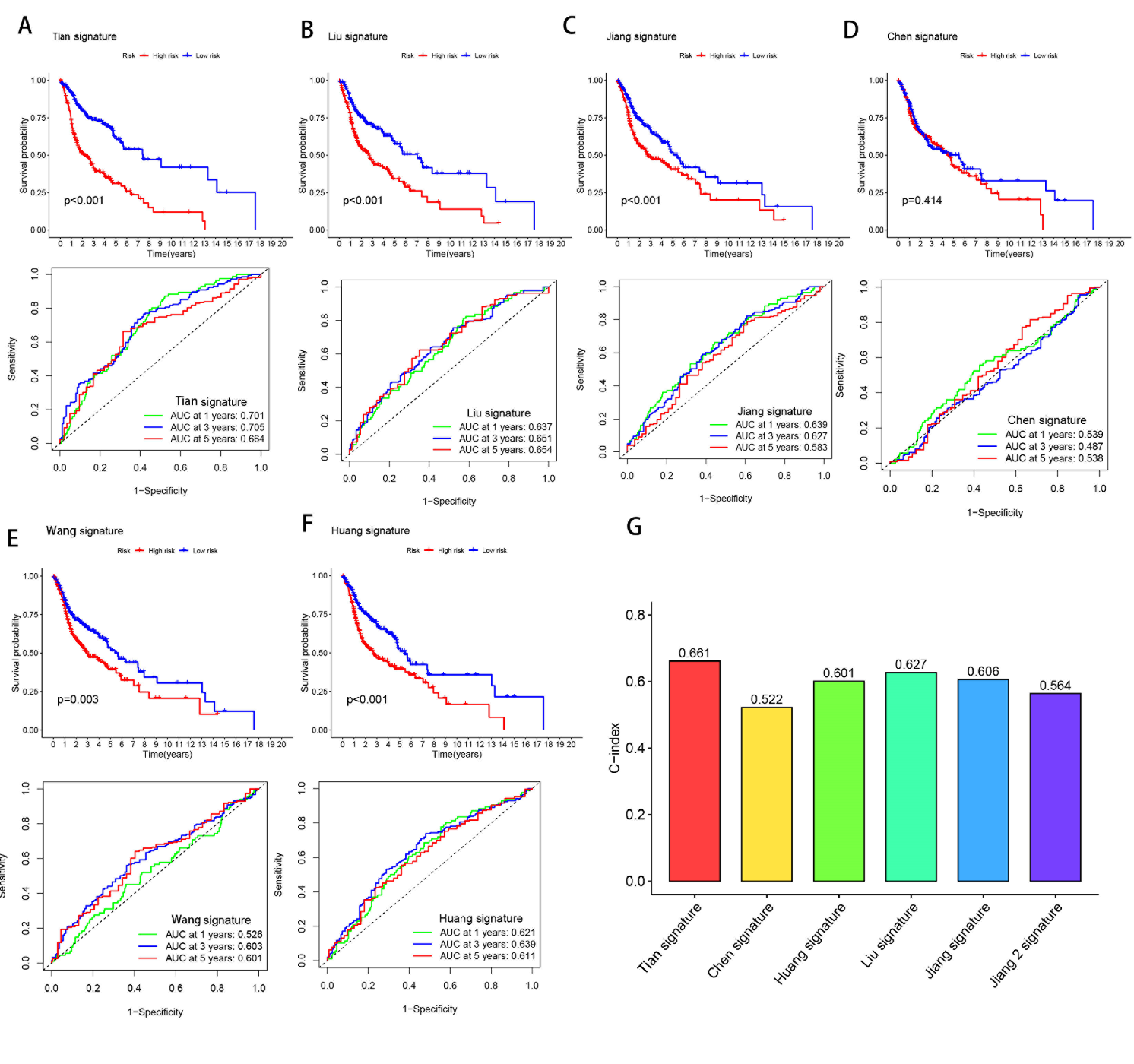


**Supplementary Figure 3** 6-CRRGs signatures have better prognostic predictive performance than other signatures. (A) KM curves and ROCs for Tian signature, (B) Liu signature, (C) Jiang signature,(D) Chen signature,(E) Huang signature,(F) and Wang signature. (G) C-index of six signatures..


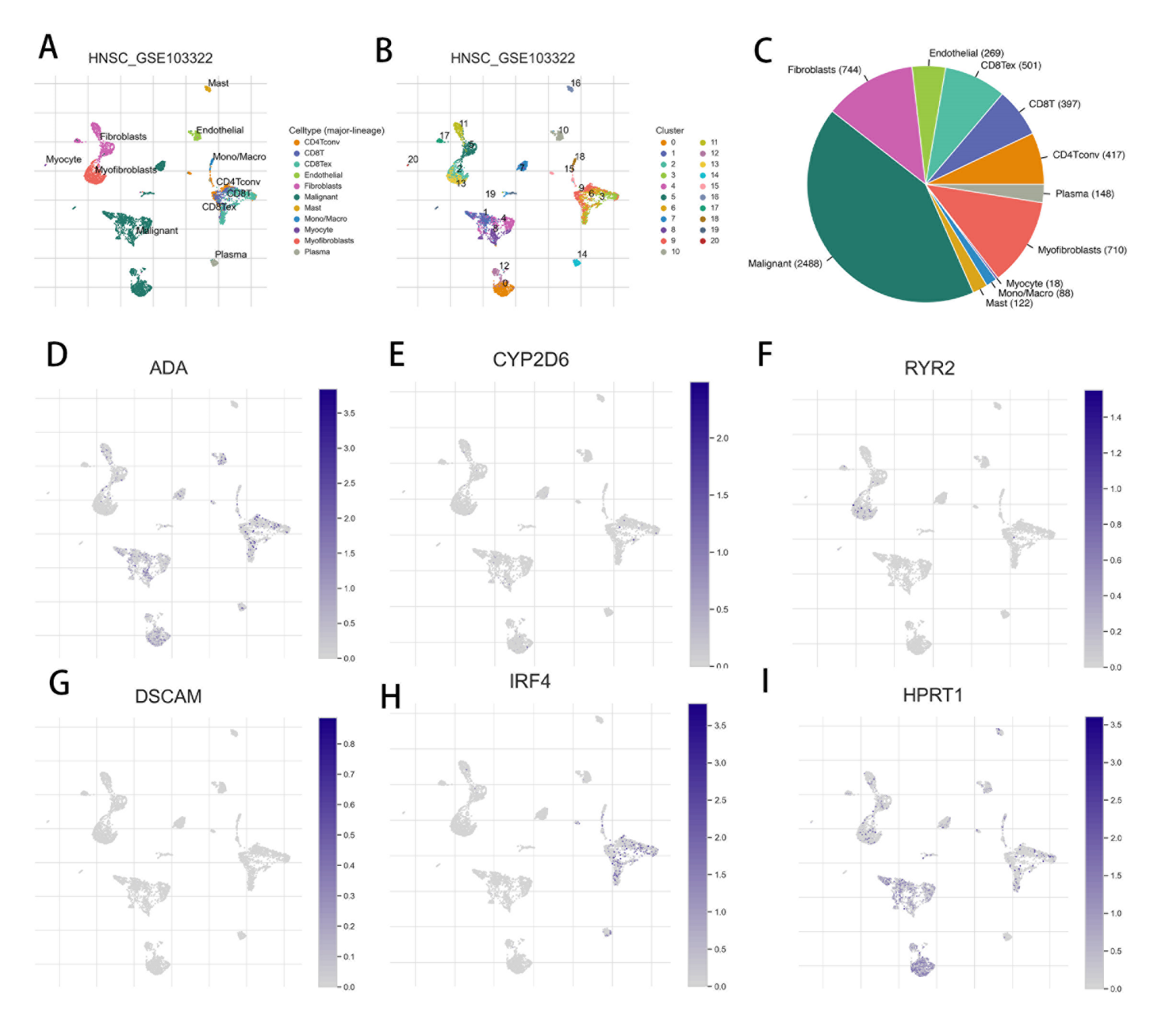


**Supplementary Figure 4** 6-CRRGs Expression in HNSCC TME-associated cells. (A-D) Annotation of all cell types in GSE103322 and the percentage of each cell type. (E) Percentages and expressions of 6-CRRGs.
